# Methanol and ethanol modulate responses to danger- and microbe-associated molecular patterns

**DOI:** 10.3389/fpls.2014.00550

**Published:** 2014-10-15

**Authors:** Claire T. Hann, Carlton J. Bequette, James E. Dombrowski, Johannes W. Stratmann

**Affiliations:** ^1^Department of Biological Sciences, University of South CarolinaColumbia, SC, USA; ^2^National Forage Seed Production Research Center, United States Department of Agriculture – Agricultural Research ServiceCorvallis, OR, USA

**Keywords:** methanol, ethanol, MAMP, DAMP, MAPK, oxidative burst, systemin, flg22

## Abstract

Methanol is a byproduct of cell wall modification, released through the action of pectin methylesterases (PMEs), which demethylesterify cell wall pectins. Plant PMEs play not only a role in developmental processes but also in responses to herbivory and infection by fungal or bacterial pathogens. Molecular mechanisms that explain how methanol affects plant defenses are poorly understood. Here we show that exogenously supplied methanol alone has weak effects on defense signaling in three dicot species, however, it profoundly alters signaling responses to danger- and microbe-associated molecular patterns (DAMPs, MAMPs) such as the alarm hormone systemin, the bacterial flagellum-derived flg22 peptide, and the fungal cell wall-derived oligosaccharide chitosan. In the presence of methanol the kinetics and amplitudes of DAMP/MAMP-induced MAP kinase (MAPK) activity and oxidative burst are altered in tobacco and tomato suspension-cultured cells, in *Arabidopsis* seedlings and tomato leaf tissue. As a possible consequence of altered DAMP/MAMP signaling, methanol suppressed the expression of the defense genes *PR-1* and *PI-1* in tomato. In cell cultures of the grass tall fescue (*Festuca arundinacea*, Poaceae, Monocots), methanol alone activates MAPKs and increases chitosan-induced MAPK activity, and in the darnel grass *Lolium temulentum* (Poaceae), it alters wound-induced MAPK signaling. We propose that methanol can be recognized by plants as a sign of the damaged self. In dicots, methanol functions as a DAMP-like alarm signal with little elicitor activity on its own, whereas it appears to function as an elicitor-active DAMP in monocot grasses. Ethanol had been implicated in plant stress responses, although the source of ethanol in plants is not well established. We found that it has a similar effect as methanol on responses to MAMPs and DAMPs.

## INTRODUCTION

Plants release methanol as a volatile compound, sometimes in relatively large quantities (Fall and Benson, 1996). Most plant-derived methanol is generated by pectin methylesterases (PMEs). PME releases methanol as a byproduct during demethylesterification of the homogalacturonan (HG) component of cell wall pectins. Other sources of methanol from plants, such as DNA demethylation and protein repair pathways, have been postulated but seem to have a minor contribution to overall emissions (Fall and Benson, 1996; Oikawa et al., 2011). The volatility of methanol allows it to rapidly exit leaf tissue via stomata. In dicots, pectins can represent more than 30%, and in monocots about 10% of the total cell wall polysaccharides. HG polymers are the most abundant pectins (Lionetti et al., 2012). The D-galacturonic acid residues of pectin are methyl-esterified during synthesis in the Golgi, and demethylesterification of these residues by PMEs *in muro* exposes negatively charged carboxyl groups to Ca^2+^ ions. Ca^2+^ acts as a crosslinker between HG polymers affecting wall properties such as porosity, stiffening, and cell adhesion (Caffall and Mohnen, 2009; Lionetti et al., 2012). Demethylesterification of HG is an essential part of cell wall maturation, which results in strengthening of the mature cell wall. As a consequence, young leaves exhibit relatively high PME activities and methanol emissions, while methanol emissions from mature leaves are much lower (MacDonald and Fall, 1993; Nemecek-Marshall et al., 1995; Oikawa et al., 2011). At the same time, the activity of PME can also increase cell wall fluidity by exposing the glycosidic bonds of demethylesterified HG to polygalacturonases and pectic lyases, which cleave the pectin polymer (Lionetti et al., 2012).

The plant cell wall is not only important for developmental processes, but also represents a primary physical barrier against pathogens like bacteria and fungi. Cellulose makes up about 30% of the cell wall. Its tightly packed microfibril arrangement makes it difficult to penetrate, leaving pectin as the prime target of pathogens. During infections, the plant cell wall matrix is turned into a battlefield where pathogens deploy an arsenal of hydrolytic enzymes, including cutinases, lipases, carbohydrate esterases, and polysaccharide lyases (van den Brink and de Vries, 2011; Bellincampi et al., 2014). In a tit for tat, plants respond with mobilization of hydrolytic enzymes that attack the cell walls of pathogens such as chitinases and beta-glucanases. Demethylesterification of HGs is an important strategy for both plants and pathogens as it makes HGs accessible to polygalacturonases, which break down HG to oligogalacturonide fragments (OGAs).

Although the OGAs produced by polygalacturonases are derived from plant HGs, their presence is indicative of cell wall damage due to an ongoing attack. Plants can recognize OGAs as a sign of their “damaged-self” resulting in the launch of a counter defense response (Heil, 2009, 2012). Therefore, the OGAs function as elicitors of plant defenses and classical danger- or damage-associated molecular patterns (DAMPs). In order for OGAs to function as DAMPs, they must be demethylesterified by PMEs to be active (Lionetti et al., 2012). Both methanol and OGAs are not only released through the action of enzymes from pathogens, but also during cell wall maturation processes through the action of plant PMEs and polygalacturonases. Moreover, OGAs also function as developmental signals (Wolf et al., 2012). Therefore, when it comes to plant defenses, it is critically important for plants to filter out the “developmental noise” of cell wall breakdown products that are not indicative of pathogens. In tomato leaves, an endogenous polygalacturonase is expressed in response to wound signals, presumably as an amplification process to generate high levels of OGAs to favor DAMP function (Bergey et al., 1999). Examples for additional plant DAMPs are extracellular ATP and sucrose (Tanaka et al., 2010; Heil et al., 2012), and small peptides like the alarm hormone systemin (Pearce et al., 1991), hydroxyproline-rich systemins (Pearce et al., 2001; Pearce, 2011) and plant elicitor peptides (Peps; Huffaker et al., 2006). In contrast, microbe-associated molecular patterns (MAMPs) are not found in plants. They are characteristic of relatively large phylogenetic groups of bacteria, fungi, or oomycetes. Examples for MAMPs relevant for the investigation presented here are flg22, a 22 amino acid peptide from the flagellin protein, the main constituent of bacterial flagella (Felix et al., 1999) and chitosan, a non-acetylated polyglucosamine from fungal cell walls (Bartnicki-Garcia, 1968), which is derived from chitin (poly-*N*-acetylglucosamine) via deacetylation by chitin deacetylases (El Gueddari et al., 2002; Baker et al., 2007). Recognition of flg22 and chitosan would enable plants to recognize all motile bacteria and most fungi, at least in principle. MAMPs and DAMPs are typically perceived by membrane-bound receptors, which elicit plant defense responses via a complex signaling network which includes MAP kinase (MAPK) cascades and an oxidative burst of reactive oxygen species (ROS; Boller and Felix, 2009; Newman et al., 2013).

Pectin methylesterases have been implicated in plant resistance against pathogens and herbivores, but only a few studies investigated the direct effects of methanol on plant defenses. PMEs are of critical importance for virulence. A higher degree of cell wall methylation correlates with disease resistance in multiple plant species (Lionetti et al., 2007; Reignault et al., 2008; Raiola et al., 2011; Volpi et al., 2011; Lionetti et al., 2012). The infection process also regulates the expression of certain plant PMEs, thereby altering the outcome of plant-pathogen interactions (Lionetti et al., 2012). The necrotrophic pathogen *Pectobacterium carotovorum* induces *Arabidopsis* PME3, resulting in pectin demethylesterification and increased susceptibility to the pathogen. So the pathogen manipulates and exploits a plant PME to render the plant cell wall susceptible to attack by its own cell wall degrading enzymes (Raiola et al., 2011). In the Banana-*Fusarium* pathosystem, different expression levels of PMEs in different banana cultivars correlate with resistance or susceptibility to the pathogens (Ma et al., 2013).

Plant PMEs also play a role in defenses against herbivores. In *Nicotiana attenuata* plants, herbivory increased leaf PME expression and activity, which resulted in increased methanol release (von Dahl et al., 2006). Körner et al. (2009) showed that *N. attenuata* plants silenced for a leaf PME produced less methanol, showed reduced insect-induced jasmonic acid (JA) and salicylic acid (SA) synthesis, reduced defense proteins and reduced resistance to herbivorous *Manduca sexta* (Lepidoptera) larvae. This is consistent with a study where PMEs were overexpressed in transgenic tobacco plants, which produced much higher levels of methanol and were more resistant against herbivores, including chewing lepidopteran larvae as well as sucking/piercing aphids and whiteflies (Dixit et al., 2013).

Direct effects of methanol in plant defenses have garnered relatively little attention. In many studies on the roles of PME or methanol a functional link between the two was not considered. There is strong evidence that methanol plays some role in plant defenses against pathogens and herbivores. Peñuelas et al. (2005) analyzed VOC emissions induced by larvae of *Euphydryas aurinia* (Lepidoptera) feeding on *Succisa pratensis* (Dipsacaceae) leaves. Methanol was emitted at much higher rates than all other detected VOCs. The methanol emission rate from damaged leaves was about sixfold higher than from undamaged leaves, but undamaged leaves still emitted relatively high amounts of methanol as compared to other VOCs. Similarly, in *N. attenuata* plants, herbivory increased leaf PME expression and activity and resulted in increased methanol release. Mimicking methanol release by exogenous application suppressed herbivory-induced accumulation of defensive proteinase inhibitor proteins and thus decreased resistance of *N. attenuata* plants to *M. sexta* larvae (von Dahl et al., 2006). In rice leaves, methanol, but not ethanol, can function as a “signal” that upregulates tryptophan-based secondary metabolism, which is involved in defenses against pathogens and herbivores. Methanol effects were regulated by cytokinin and ABA, and directly correlated with the expression levels of certain PMEs (Kang et al., 2011). An investigation of the transcriptional response of *Arabidopsis* to methanol using a whole genome microarray experiment, revealed that 1.9% of all represented transcripts responded to a treatment of 10% methanol. Upregulated transcripts fell in the categories of signaling, defense, metabolism, especially flavonoid metabolism, and detoxification (Downie et al., 2004). 10% is a relatively high methanol concentration and it remains to be determined whether methanol may accumulate to such high levels in cell wall microenvironments. The authors suggested that this represents a xenobiotic perturbation study and hypothesized that methanol induces gene expression via its electrophilic and toxic breakdown product formaldehyde.

After its release, methanol has the potential to function as a systemic intraplant or an interplant alarm signal by alerting adjacent leaves or neighboring plants of an attack. This is well known from other VOCs such as green leaf volatiles (Engelberth et al., 2004; Heil and Silva Bueno, 2007). Dorokhov et al. (2012) and Komarova et al. (2014) showed that wound-induced methanol emitted from *Nicotiana* plants increased resistance to *Ralstonia solanacearum* bacteria in unwounded conspecific receiver plants, but not in the controls that did not receive methanol. The authors also showed that methanol increases the spread of tobacco mosaic virus in *N. benthamiana* plants. These findings add a new dimension to methanol functions in plants. Taken together, it is evident that methanol is not only a byproduct of PME activity, but also affects secondary metabolism and gene expression, and it functions as an interplant alarm signal.

Ethanol is structurally similar to methanol. As a product of anaerobic fermentation it is not expected to be generated in aerial parts of plants. However, anoxic conditions such as flooding can result in ethanol fermentation and subsequent transport of ethanol to aerial parts of the plant (Kreuzwieser et al., 1999). Moreover, ethanol and acetaldehyde can be produced under aerobic stress conditions (Kimmerer and Kozlowski, 1982; MacDonald et al., 1989; Tadege et al., 1999).

It remains to be determined whether methanol and ethanol activate signaling processes that translate the methanol/ethanol signal into an output response such as gene expression. We tested whether methanol would activate MAPKs, an oxidative burst, and gene expression in a manner similar to MAMPs and DAMPs (Boller and Felix, 2009). We found that methanol does not function as an elicitor of these responses in several dicot plants, however, it alters signaling and gene expression in response to the wound signaling peptide systemin (a DAMP) or the MAMPs flg22 and chitosan. In contrast, methanol strongly induces signaling responses in grasses. Ethanol had similar effects on signaling as methanol.

## MATERIALS AND METHODS

### GROWTH CONDITIONS

#### Suspension-cultured cells

*Solanum peruvianum* (Felix and Boller, 1995; Yalamanchili and Stratmann, 2002), *Nicotiana tabacum* (Bednarek et al., 1990; Pearce et al., 2001), and tall fescue (*F. arundinacea*) suspension-cultured cells were cultivated in 125 ml Erlenmeyer flasks on an orbital shaker (160 rpm) under ambient room light and temperature conditions. Tall fescue suspension-cultured cells were produced from calli induced from explant tissue derived from Tall fescue cv. Kentucky 31 endophyte-minus seeds as described (Martin et al., 2012). Cells were subcultured weekly and used for experiments 7 days after subculturing.

#### Plants

Tomato plants (*S. lycopersicum; S*. *cheesemaniae*) were grown in AR66L growth chambers (Percival Scientific, Perry, IA) on a 16 h light (130 ± 20 μE m^-2^ s^-1^; 27°C) and 8 h dark (22°C) cycle in Miracle-Gro Potting Mix. *Arabidopsis thaliana* seeds were sterilized in ethanol (1 min) followed by brief vortexing with a 30% commercial bleach solution containing 20% Triton X 100, allowed to stand for 5 min, and washed with sterile water ten times. Seeds were vernalized for 48 h and germinated on 1/2 MS plates with sucrose at 25°C day/22°C night on a 16 h light-8 h dark cycle. *L. temulentum* L. (Darnel ryegrass) cv. Ceres plants were grown as described (Dombrowski et al., 2011).

### TREATMENTS OF CELL SUSPENSIONS AND PLANTS

For experiments, 1.5 ml of cell suspensions were transferred to each well in 12-well plates (Falcon tissue culture plates – BD Biosciences, San Jose, CA, USA) that were shaken (150 rpm) on an orbital shaker under ambient room light and temperature conditions. Treatments started after a ∼1-h adjustment period. Each sample was represented by cells from two wells that were treated identically. Ethanol and methanol treatments were delivered immediately before elicitor treatments. All percentages are volume per volume (v/v). Systemin and flg22 were purchased from GenScript (Piscataway, NJ, USA) and solved in H_2_O. Chitosan was purchased from Sigma-Aldrich (St. Louis, MO, USA) and dissolved in 0.4% v/v glacial acetic acid (2.55 mg/mL). Elicitor treatment volumes ranged from 1–10 μL. Upon sampling, cells were separated from media using a Büchner funnel with Miracloth and flash-frozen in liquid nitrogen. For tall fescue assays, the transferred cells were incubated on a rotatory shaker (125 rpm) in the dark for 3–4 h. For double treatments, chitosan was added first, immediately followed by methanol. For sampling, cells were collected, centrifuged at 14,000 rpm for 15 s to remove the medium, and then flash-frozen.

*Solanum lycopersicum* and *S. cheesemaniae* leaf disks were cut using a cork borer with a diameter of 7 mm, placed adaxial side up in sterile 6-well Costar tissue culture plates (Corning, Corning, NY, USA), and incubated overnight in sterile water on a rotatory shaker (100 rpm). Each well contained eight leaf disks for one sample. Both incubation and treatment were performed in an AR66L growth chamber (65 ± 5 μE m^-2^ s^-1^; 25°C). Disks were treated by replacing the water with aqueous solutions of 3% methanol, flg22, and flg22 + methanol. Samples were collected using forceps. Leaf disks were quickly blotted to remove water and flash-frozen immediately thereafter.

*Arabidopsis* seedlings at the 4-6-leaf stage were removed from 1/2 MS plates and placed in a sterile 24-well Costar tissue culture plate (Corning, Corning, NY, USA), submersed in 2 mL Gamborg’s medium (2% sucrose with vitamins) and shaken on a rotatory shaker at 100 rpm. Eight to 10 seedlings were used per sample. After transfer, plants in wells were incubated overnight in the growth chamber before treatment to provide time for downregulation of potential stress responses caused by the transfer. Each well was treated with methanol or flg22 or a combination of methanol and flg22 in 0.5 mL of Gamborg’s medium for a final volume of 2.5 mL. For sampling, plants were quickly collected from wells with fine-tip forceps, gently blotted to remove media, and flash-frozen.

For *L. temulentum* wounding experiments, plants were sprayed with either deionized water or a 5% methanol solution in deionized water. Immediately after spraying, the plants were wounded one time perpendicular to the tiller using a hemostat below the junction of where the leaf emerges from the pseudostem.

### MAPK ACTIVITY ASSAY

Proteins from suspension cells and plants were extracted as described (Holley et al., 2003). Thirty to 50 μg total protein was analyzed for MAPK phosphorylation by immunoblotting using an anti-phospho-ERK antibody (phospho-p44/42 MAPK (Erk1/2; Thr202/Tyr204); D13.14.4E; Cell Signaling Technology) at a dilution of 1:2,500 (1:2,000 for grasses) in 5% BSA as described (Dombrowski et al., 2011). This antibody specifically recognizes active MAPKs that are dually phosphorylated on a threonine and tyrosine residue in a TEY phosphorylation motif, which is conserved among certain plant and metazoan MAPKs (Hind et al., 2010; Bethke et al., 2012). In grasses, MAPKs were detected using an alkaline phosphatase NBT/BCIP system as described (Dombrowski et al., 2011). In dicots, MAPKs were detected by X-ray film using a chemiluminescence assay (Lumi-Phos substrate, Thermo Scientific, Fisher Scientific) with a anti-rabbit IgG secondary antibody coupled to alkaline phosphatase (Sigma–Aldrich). Equal protein loading was confirmed by staining proteins on membranes with coomassie brilliant blue (CBB). MAPK signals on immunoblots were quantified using ImageQuant TL software (GE Healthcare).

### OXIDATIVE BURST ASSAY

Ninety-six leaf disks from *S. lycopersicum* or *S. cheesemaniae* were cut using a cork borer with a diameter of 4 mm. One disk was placed adaxial side up into each well of a 96-well plate (Lumitrac 200; Greiner Bio-One, Monroe, NC, USA) and incubated in water overnight (ambient room temperature and light).

Controls (untreated, methanol) were represented with 16 disks per plate, treatments with elicitor and elicitor plus methanol were represented by 32 disks per plate each. Immediately before treatment, the water was removed and the wells were filled with an aqueous solution containing 34 μg/mL luminol (Sigma–Aldrich) and 20 μg/mL horseradish peroxidase (MP Biomedicals, Santa Ana, CA, USA) each, plus 10 nM flg22, 3% methanol, flg22 + methanol, or water (untreated). Immediately following treatment, chemiluminescence was measured for 60 min, with readings taken every 64 s, using a Synergy HT Multi-Mode Microplate Reader (BioTek, Winooski, VT, USA) in Kinetic Read Mode, set to read endpoint luminescence in relative luminescence units (RLU). Data were collected using Gen5 Data Analysis Software (BioTek).

### RNA EXTRACTION AND GENE EXPRESSION ANALYSIS

For reverse transcription (RT) quantitative real-time PCR (qPCR), total RNA was extracted from tomato leaf disks (∼50 mg) using TRIzol®; reagent (Life Technologies, Grand Island, NY, USA). RNA was quantified using a SmartSpec Plus spectrophotometer (Bio-rad, Hercules, CA, USA) and confirmed by electrophoresis using a 1% agarose gel. Extracted RNA was treated with Turbo DNase (Life Technologies). First strand cDNA was synthesized using 1 μg of RNA with iScript^TM^ RT Supermix for RT-qPCR (Biorad). The protocol for the synthesis of cDNA was: priming (25°C, 5 min), RT (42°C, 30 min), and RT inactivation (85°C, 5 min). The cDNA was diluted 1:30 for gene expression analysis via qPCR. RT-qPCR was carried out using a Bio-rad C1000 and SsoAdvanced Universal SYBR Green Supermix (Bio-rad). The protocol for RT-qPCR was: 95°C for 30 s, followed by 50 cycles of 95°C for 15 s and 55°C for 30 s. The melting curves were analyzed at 55–95°C for 80 cycles (0.5°C increments for 5 s). Primers used for RT-PCR: *PR1a* (forward: GAGGGCAGCCGTGCAA; reverse: CACATTTTTCCACCAACACATTG; 81 bp amplification product), *PI-1* (forward: GAAACTCTCATGGCACGAA; reverse: GATGGATTTTCCTTCTCAATTATTTCC; 162 bp), *18 s* (forward: GTCCAGACATAGTAAGGATTGA; reverse: TAACCAGACAAATCGCTCCA; 101 bp). Accession numbers: *PR-1a (P4)*-AJ011520 or CAA09671.1 or M69247.1; *PI-I*-AAA60745.1 or AAA34200.1 or M13938.1; *18S-rRNA*-X51576.1. Primer specificity was confirmed by melting curve analysis for every reaction. Non-reverse transcribed samples were run as a control for each RT-qPCR run. Three technical replicates were used for each sample during every RT-qPCR run. Average C_T_ and primer efficiencies were determined using Real-time PCR Miner (; Zhao and Fernald, 2005). Fold difference in target gene expression was calculated using the Pfaﬄ method (Pfaﬄ, 2001) and *18 s-rRNA* as the reference gene.

## RESULTS

### METHANOL AND ETHANOL ACTIVATE PLANT MAP KINASES

We tested whether methanol and ethanol would signal through MAPKs, a characteristic early signaling response to DAMPs and MAMPs. MAPK activity was measured by immunoblotting with an antibody specific for active MAPKs (Hind et al., 2010; Bethke et al., 2012). In tomato (*S. lycopersicum*) the major stress responsive MAPKs are MPK1, MPK2, and MPK3. Orthologs in the wild tomato species *S. peruvianum* are almost identical. MPK1 and 2 are products of a recent gene duplication event and 95% identical at the amino acid level (Holley et al., 2003; Kandoth et al., 2007). They are functionally redundant, have the same size (∼48 kDa on immunoblots), and thus cannot be distinguished by immunoblotting using antibodies against active MAPKs. In tobacco (*N. tabacum*), the orthologs of MPK1, 2, and 3 are SIPK, NTF4, and WIPK, respectively, (Ren et al., 2006). In *Arabidopsis*, the single ortholog of MPK1/2 or SIPK/NTF4 is AtMPK6, and the ortholog of MPK3/WIPK is AtMPK3 (Holley et al., 2003). On immunoblots, AtMPK6 and orthologs separate with an apparent MW of ∼48 kDa, and AtMPK3 and orthologs with an apparent MW of ∼44 kDa. Some stress conditions activate a third smaller MAPK, which is likely an ortholog of *Arabidopsis* MPK4 and MPK11, two paralogs that are 88% identical (92% similar) at the amino acid level (Bethke et al., 2012). We also assayed MAPK activity in the grasses tall fescue (*F. arundinacea*) and darnel ryegrass (*L. temulentum*; both Poaceae, Monocotyledons). While the pattern of active MAPK bands on immunoblots is similar to dicotyledonous plants, the exact identity of these grass MAPKs is unknown (Dombrowski et al., 2011). However, MAPKs from rice (also Poaceae) are well characterized and orthologs of AtMPK3, 4 and 6 have been reported (Hamel et al., 2006). To simplify naming of MAPK orthologs in various species in this text, we will use the *Arabidopsis* nomenclature for all dicot species.

**FIGURE 1:**
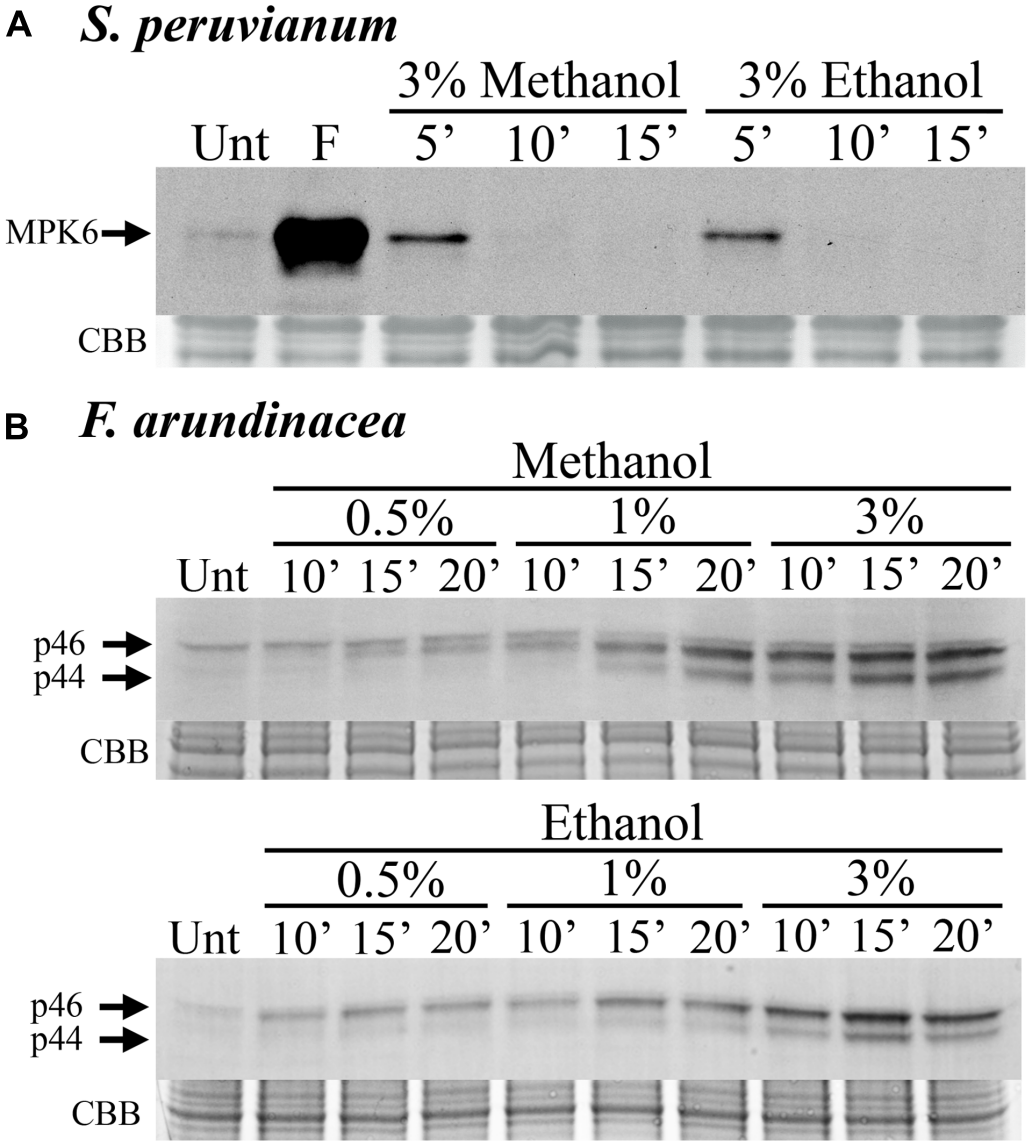
**Methanol and ethanol activate MAP kinases (MAPKs) in dicot and monocot cell lines. (A)** *Solanum peruvianum* suspension-cultured cells were treated for 5, 10, or 15 min with 3% (v/v) methanol or ethanol, and analyzed for MAPK activity by immunoblotting using an anti-phosphoERK antibody that specifically detects phosphorylated active MAPKs. The arrow indicates MPK6 activity. Unt: untreated; F: flg22 at 15 min, positive control. A representative experiment is shown. The experiment was repeated three times (methanol) or two times (ethanol) with similar results. **(B)** *Festuca arundinacea* suspension-cultured cells were treated for 10, 15, and 20 min with 0.5, 1, or 3% (v/v) methanol or ethanol and analyzed for the activity of two grass MAPKs, p46 and p44, as described in **(A)**. Unt: untreated. Representative experiments are shown. The Methanol and ethanol experiments were repeated three and two times, respectively, with similar results. Equal protein loading was confirmed by staining proteins on membranes with coomassie brilliant blue (CBB).

In *S. peruvianum* suspension-cultured cells, 3% (v/v) methanol and ethanol induced very weak and transient MPK6 activity. The activity is only detectable at 5 min after treatment and returns to background levels by 10 min (**Figure 1A**). In *N. tabacum* suspension-cultured cells, the kinetics of methanol-induced MPK6 activity were very similar (data not shown). In contrast, in *F. arundinacea* suspension-cultured cells, 0.5%, 1%, and 3% methanol and ethanol induced a relatively strong and prolonged MAPK response, which lasted longer than 20 min (**Figure 1B**). This response involves two MAPKs with apparent MWs of ∼46 (p46 MAPK) and ∼44 kDa (p44 MAPK). A third (∼47 kDa) band did not change in response to methanol or chitosan and was not considered. These data indicate that methanol activates MAPKs in grasses (monocots) and dicots, but with different activation kinetics.

### METHANOL AND ETHANOL MODULATE DAMP- AND MAMP-INDUCED MAPK ACTIVITY

**FIGURE 2:**
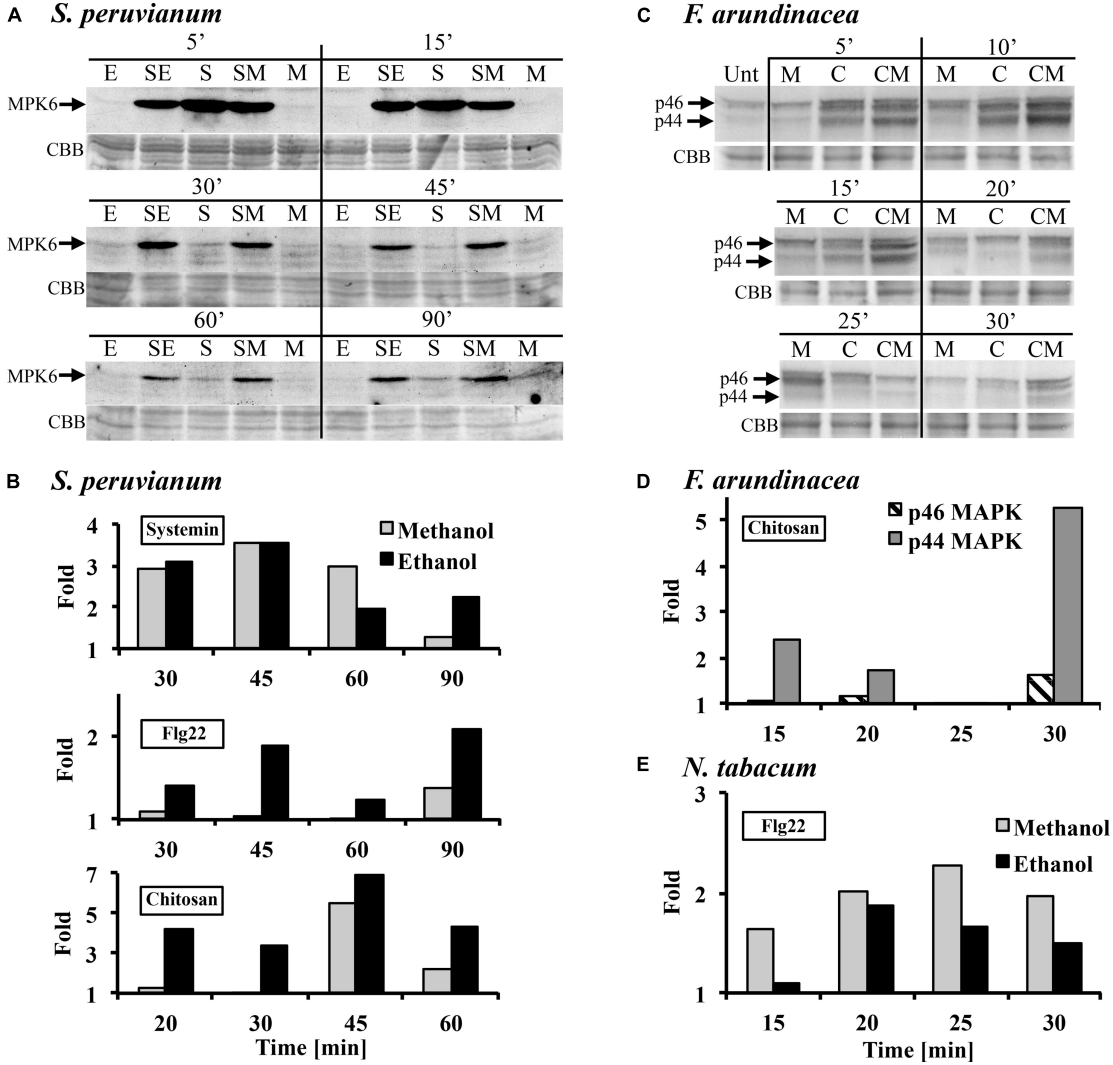
**Methanol and ethanol modulate damage-associated molecular pattern (DAMP)- and microbe-associated molecular pattern (MAMP)-induced MAPK activity. (A)** *S. peruvianum* cells were treated for the times indicated with either 3% (v/v) ethanol alone (E), 3% (v/v) methanol alone (M), 3.3 nM systemin alone (S), or a combination of either 3% ethanol and 3.3 nM systemin (SE), or 3% methanol and 3.3 nM systemin (SM). Cells were analyzed for MPK6 activation as described in **Figure 1**. Equal protein loading was confirmed by staining proteins on membranes with CBB. **(A)** representative experiment is shown. A quantification is shown in **(B)**. **(B)** Experiments were carried out as in **(A)** testing the effect of methanol and ethanol on MPK6 activity induced by either 3.3 nM systemin, 7 nM flg22, or 1.7 μg/mL chitosan. MPK6 signals on immunoblots were quantified using ImageQuant software. Only synergistic effects of the double treatments are shown in terms of fold quantities, which were calculated as follows: Combined treatment (SE, SM) divided by [M or E + elicitor alone]. Representative experiments are shown. The experiment was repeated three (flg22) or two times (systemin, chitosan) with similar results. **(C)** *F. arundinacea* cells were treated for the times indicated with 1% (v/v) methanol alone (M), 17 μg/mL chitosan alone **(C)**, or a combination of 1% methanol and chitosan (CM). Cells were analyzed for the activation of two grass MAPKs, p46 and p44, as described in **Figure 1**. Equal protein loading was confirmed by staining proteins on membranes with CBB. A representative experiment is shown. The experiment was repeated two times with similar results. **(D)** Quantification of the immunoblot shown in **(C)**, as described in **(B)**. **(E)** *N. tabacum* cells were treated for the times indicated with 3% (v/v) methanol alone, 300 nM flg22 alone, or a combination of methanol and flg22. Quantification of the immunoblot was as described in **(B)**. A representative experiment is shown. The experiment was repeated three times with similar results.

In tomato and tobacco cells, the MAPK response to ethanol and methanol is very weak (**Figure 1A**). Therefore, we tested whether the presence of methanol would alter the response to the peptide DAMP systemin, the peptide MAMP flg22, and the oligosaccharide MAMP chitosan. When *S. peruvianum* cells were incubated simultaneously with 3% methanol or ethanol and systemin or flg22 or chitosan, the MPK6 response to the elicitors was altered (**Figure 2**). During the first 15 min after treatment, the response to the double treatment was either slightly reduced or unaltered as compared to the elicitor alone treatment. Starting at around 30 min after double treatment with methanol or ethanol and one of the three elicitors, the MAPK response to the double treatment was clearly stronger than the response to the elicitor alone, and this effect lasted until 90 min or longer after treatment (**Figures 2A,B**). Since methanol- or ethanol-induced MPK6 activity had decreased to background levels by 10 min, the effect of the double treatment was synergistic, i.e., double treatments induced MAPK activity to higher levels than the sum of ethanol/methanol alone plus elicitor alone treatments. The bars in **Figures 2B,D,E** show these synergistic effects. Methanol had the strongest effect on systemin, while ethanol affected all three elicitors similarly. In additional experiments only one time point (45 min for flg22 and systemin; 30 min for chitosan) was analyzed. The synergism between methanol and flg22 was observed in six of those experiments, between methanol and systemin in three, and between methanol and chitosan in two experiments. While the synergistic effect of the double treatment was reproducible at 45 min (flg22, systemin) and 30 min (chitosan), there was variability with regard to the duration and intensity of the synergistic effect, preventing a meaningful statistical evaluation. Therefore, for this and all following experiments, we mentioned in the figure legends how many biological replicates with similar results were performed.

Tobacco cells are insensitive to systemin and less sensitive than tomato to flg22. However, a double treatment of 300 nM flg22 and 3% methanol resulted in a similar MPK6 response pattern as in *S. peruvianum* cells, except for a shorter duration of the synergistic double treatment effect (up to 30 min after treatment; **Figure 2E**). Chitosan was not tested.

In tall fescue cells, a similar effect was observed with 17 μg/mL chitosan (**Figures 2C,D**). 1% methanol alone induced p44/46 MAPK activity similarly as shown in **Figure 1B**. Chitosan alone induced transient MAPK activity. The response of the p46 MAPK to double treatment with methanol and chitosan was only synergistic at 30 min after treatment, when compared to chitosan and methanol alone. A stronger synergistic response was observed for p44 MAPK at 5, 10, 15, 20, and 30 min after treatment. Two additional experiments with different methanol and chitosan concentrations also showed synergistic effects for p44 in double treatments (3% methanol + 1.7 μg/mL chitosan at 10 min; 1% methanol + 7 μg/mL chitosan at 10, 15, 20 min). Systemin is inactive and flg22 only weakly active in tall fescue. Therefore, they were not tested.

### METHANOL AND ETHANOL ENHANCE DAMP- AND MAMP-INDUCED MAPK ACTIVITY IN A CONCENTRATION-DEPENDENT MANNER

**FIGURE 3:**
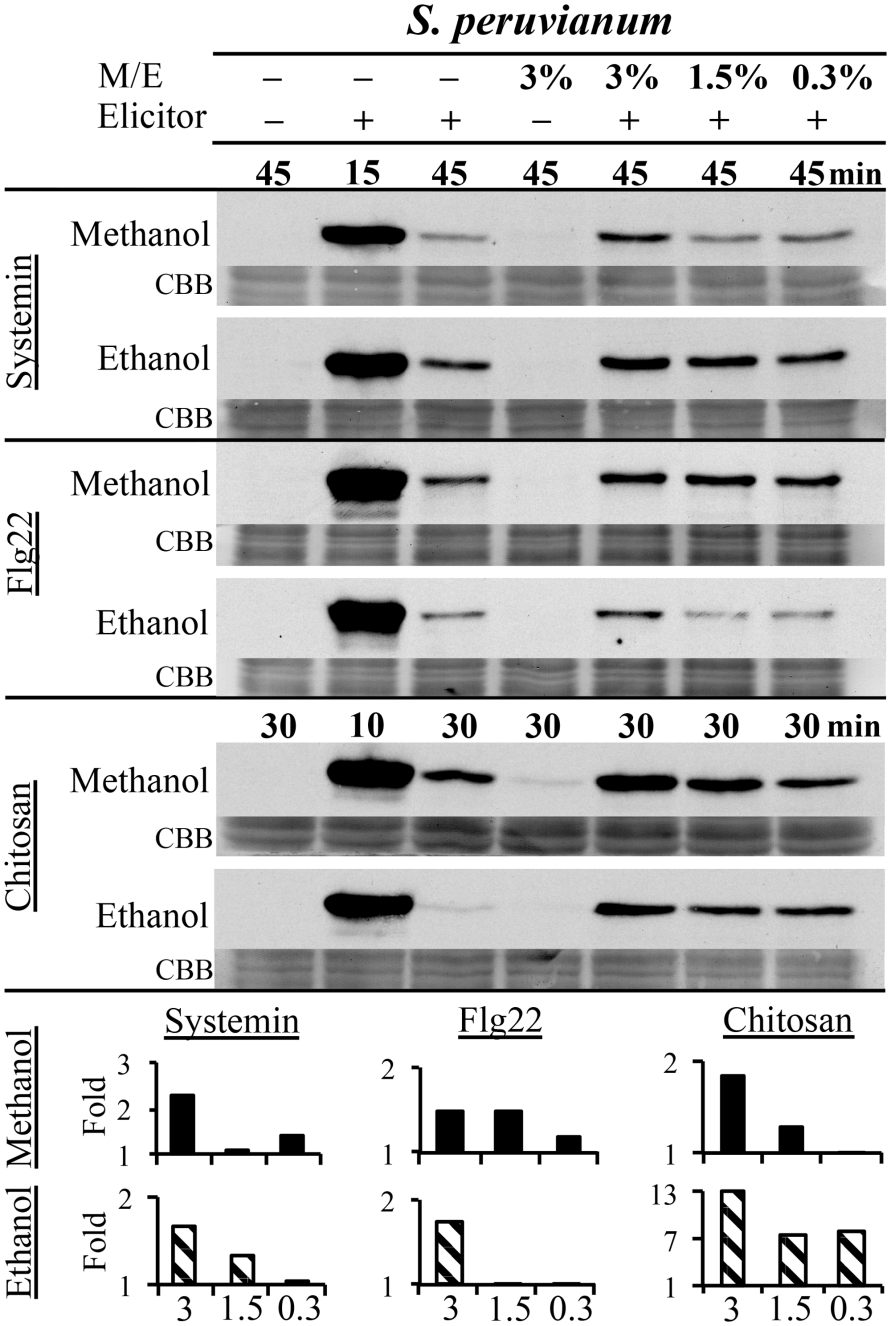
**Methanol and ethanol enhance DAMP- and MAMP-induced MAPK activity in a concentration-dependent manner.** *S. peruvianum* cells were left untreated (first column); treated for 45 or 30 min with 3% (v/v) ethanol or 3% (v/v) methanol alone (fourth column); treated for 15 min (second column; positive control) and 45 min (third column) with systemin (3.3 nM) or flg22 (7 nM) alone, or for 10 or 30 min with chitosan (1.7 μg/mL) alone; and treated with a combination of the three elicitors with either 3% (column 5), 1.5% (column 6), or 0.3% (column 7) methanol or ethanol for 45 min (systemin, flg22) or 30 min (chitosan). Cells were analyzed for MPK6 activity as described in **Figure 1**. Equal protein loading was confirmed by staining proteins on membranes with CBB. Bar graphs show quantification of synergistic MPK6 responses on the immunoblots shown, as described in **Figure 2B**. A typical experiment is shown. Reproducibility of the results is shown in **Table 1**.

Danger-associated molecular patterns and MAMPs typically elicit defense and signaling responses in a concentration-dependent manner (Pearce et al., 1991; Stratmann and Ryan, 1997; Felix et al., 1999; Gomez-Gomez et al., 1999). We tested the response to a double treatment of either flg22, systemin, or chitosan and increasing concentrations of methanol or ethanol in *S. peruvianum* cells at 45 min (systemin, flg22) or 30 min (chitosan) after treatment (**Figure 3**). At these times, both 3% methanol and ethanol did not or only very weakly activate MPK6 when supplied alone. There was response variability between double treatments, and **Table 1** shows the number of experiments that show a synergistic effect of the double treatment in relation to the number of experiments performed. The magnitude of the synergism did not always correlate with the ethanol and methanol concentrations. Therefore, **Table 1** only shows whether a synergistic effect was observed or not. The synergistic effect of ethanol on MAPK-inducing activity of all three elicitors required an ethanol concentration of above 0.3–0.6% in most experiments. Ethanol concentration-dependence was more pronounced for chitosan. For methanol, a concentration of 2.5–3% was required in most experiments for a synergistic effect with flg22. Methanol acted synergistically with systemin at all concentrations tested. The effect of methanol on chitosan was less clear, with only half of the experiments showing a synergistic effect for 2.5–3% methanol on chitosan-induced MAPK activity, and only a third of all experiments for lower methanol concentrations.

**Table 1:** Synergistic effects of ethanol and methanol on elicitor-induced MPK6 activity in *Solanum peruvianum* cells.

Elicitor	flg22	flg22	flg22	Sys	Sys	Sys	Chit	Chit	Chit
**Ethanol(v/v %)**	**2.5–3%**	**1.5–2%**	**0.3–0.6%**	**2.5–3%**	**1.5–2%**	**0.3–0.6%**	**2.5–3%**	**1.5–2%**	**0.3–0.6%**
Experiments^1^	6	6	5	5	5	5	4	4	4
Synergism observed^2^	5	5	1	5	5	3	4	1	1
**Methanol(v/v %)**	**2.5–3%**	**1.5–2%**	**0.3–0.6%**	**2.5–3%**	**1.5–2%**	** 0.3–0.6%**	**2.5–3%**	**1.5–2%**	**0.3–0.6%**
Experiments^1^	15	8	8	9	6	6	10	6	6
Synergism observed^2^	9	2	4	7	3	5	5	2	2

*Sys, systemin; Chit, chitosan; ^1^Number of experiments performed; ^2^Number of experiments in which synergistic effect of methanol/ethanol on elicitor-induced MPK6 activity was observed.*

We did not test a concentration-dependence for synergistic MAPK activation in tall fescue cells, but methanol and ethanol alone strongly induced p44 and p46 MAPK activity in a concentration-dependent manner (**Figure 1B**).

### METHANOL MODULATES FLG22- AND WOUNDING-INDUCED MAPK ACTIVITY IN LEAF TISSUE

**FIGURE 4:**
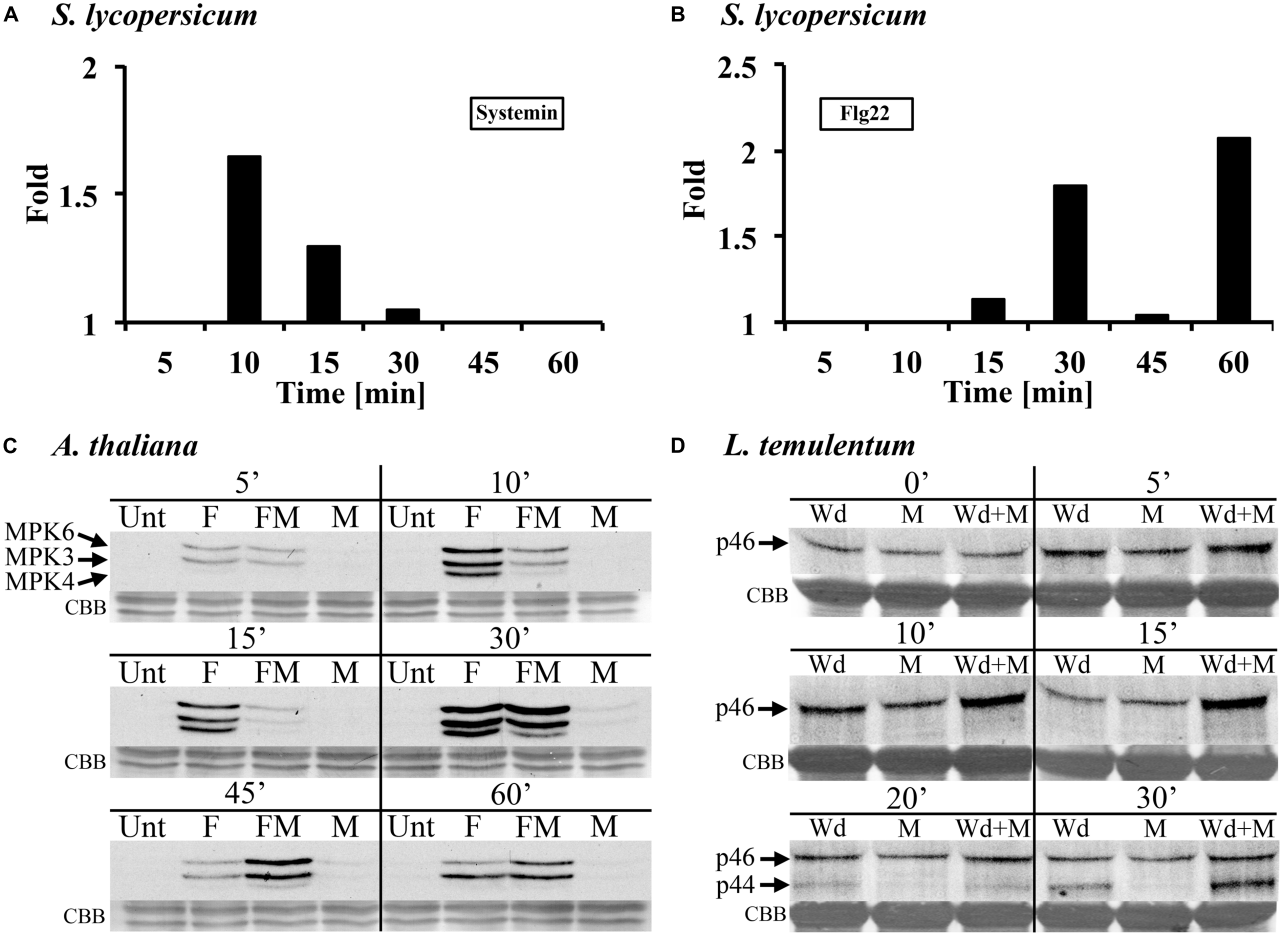
**Methanol modulates MAMP/DAMP- and wounding-induced MAPK activity in leaf tissue of tomato, *Arabidopsis*, and darnel ryegrass. (A,B)** Tomato (*S. lycopersicum* var. Rio Grande) leaf disks were treated with 3% (v/v) methanol and 25 nM systemin **(A)** or 10 nM flagellin **(B)** and analyzed for MPK6 activity as described in **Figure 1**. The experimental setup is as described for *S. peruvianum* cells in **Figure 2**. MPK6 signals on immunoblots were quantified and synergistic effects of the double treatments are shown and calculated as described in **Figure 2B**. A representative experiment is shown. Each experiment was repeated three times with similar results. **(C)** *Arabidopsis* seedlings were left untreated (Unt), treated with 3% (v/v) methanol (M), 10 nM flg22 (F), or both methanol and flg22 (FM). The experimental setup is as described in **Figure 2**. At the times indicated, seedlings were analyzed for MPK3, MPK4, and MPK6 activity as described in **Figure 1**. Equal protein loading was confirmed by staining proteins on membranes with CBB. A representative experiment is shown. The experiment was repeated six times with similar results. **(D)** Whole *Lolium temulentum* plants were either sprayed with water and tillers were wounded immediately thereafter (Wd), or sprayed with 5% (v/v) methanol alone (M), or sprayed with 5% methanol and wounded immediately thereafter (Wd + M). At the times indicated, the plants were analyzed for p44 and p46 MAPK activity as described in **Figure 2**. Equal protein loading was confirmed by staining proteins on membranes with CBB. A representative experiment is shown. The experiment was repeated three times with similar results.

All previous experiments were carried out in suspension-cultured cells. We then tested for methanol effects in leaf tissue and whole plants. In leaf disks of the tomato variety Rio Grande, we found that 3% methanol altered flg22- and systemin-induced MPK6 signaling (**Figure 4**). Methanol acted synergistically with systemin early after treatment (10–30 min; **Figure 4A**), whereas the strongest effect with flg22 was observed at later times (30 to 60 min; **Figure 4B**). Leaf disks of the Castlemart variety showed a similar response to double treatments in three out of four experiments (data not shown).

In submerged *Arabidopsis* seedlings, flg22-induced MAPK activation was altered by 3% methanol in a complex way (**Figure 4C**). Flg22 induced AtMPK6, AtMPK3, and AtMPK4. Methanol alone had almost no effect on MAPK activity at 5–60 min after application. A representative experiment is shown in **Figure 4C**. The experiment was repeated six times. In these six experiments, flg22-induced MPK6 activity in the presence of methanol was suppressed or unaltered at 10 and 15 min, however, at 30, 45, and 60 min, MPK6 activity was stronger as compared to flg22 alone (six out of six experiments for 30 and 45 min). Flg22-induced MPK3 activity was suppressed by methanol at 10 min, suppressed or unaltered at 15 min, and unaltered or stronger at 30 and 45 min, as compared to flg22 alone. At 60 min the response was highly variable. Flg22-induced MPK4 activity was suppressed by methanol at 10 and 15 min (five out of six experiments), unaltered or stronger at 30 min (in five of six experiments, but suppressed in the experiment shown in **Figure 4C**) and at 45 min (four out of six experiments), and unaltered or not detectable at 60 min, as compared to flg22 alone. These results show that, in *Arabidopsis* seedlings, methanol alters the kinetics and amplitude of three different flg22-induced MAPKs.

In the darnel ryegrass *L. temulentum*, we tested the MAPK response to wounding in the presence of methanol and ethanol. When 3–4-week-old plants were wounded once across the leaf, there was a brief and transient MAPK response (**Figure 4D**). Wounding the leaf three times results in a stronger MAPK response (Dombrowski et al., 2011). However, when plants were first sprayed with methanol and then immediately wounded, the p46 MAPK response was synergistic at 10 and 15 min. At 20 and 30 min, wounding activated a p44 MAPK, and at 30 min, the p44 response to methanol plus wounding was synergistic. When plants were sprayed with ethanol, no synergistic effect on wound-induced MAPK activity was found (data not shown).

These data show that methanol alters elicitor- and wound-induced MAPK signaling in both suspension-cultured cells and leaf tissue of monocot and dicot plants, although the specifics of the MAPK response pattern vary between systems.

### METHANOL AND ETHANOL MODULATE THE FLG22-INDUCED OXIDATIVE BURST IN LEAF TISSUE

**FIGURE 5:**
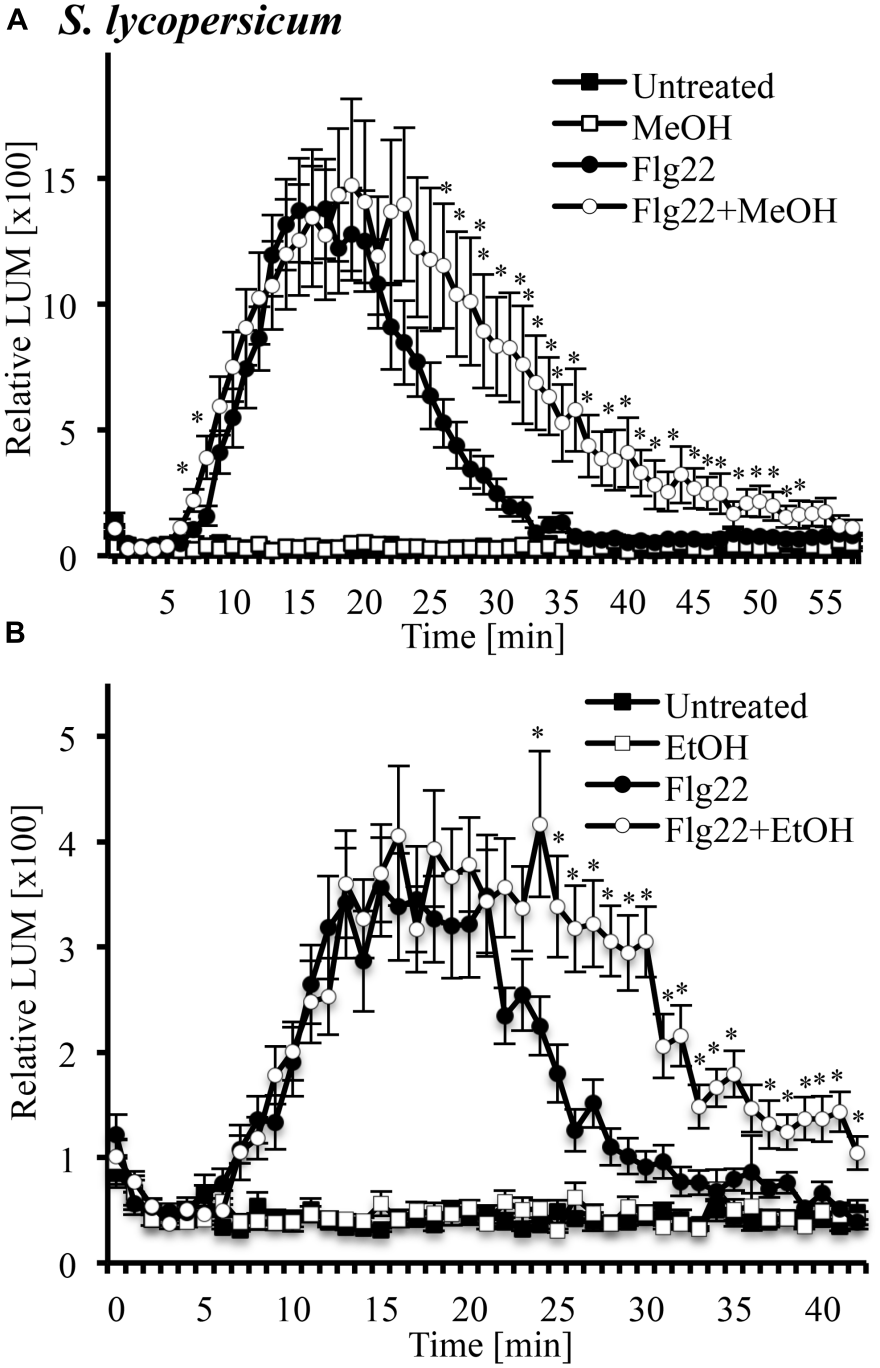
**Methanol and ethanol modulate the flg22-induced oxidative burst in leaf tissue of tomato.** Tomato (*S. lycopersicum* var. Rio Grande) leaf disks were left untreated, treated with 3% (v/v) methanol alone (MeOH; **A**), 3% ethanol alone (EtOH; **B**), 10 nM flg22 alone, or with a combination of methanol/ethanol and flg22 (Flg22 + MeOH and Flg22 + EtOH) and analyzed for accumulation of reactive oxygen species (ROS) at the times indicated using a chemiluminescence assay. Luminescence was measured at 64 s intervals. Data represent the average of eight individual leaves with four or two technical replicates each ±SE for treatments and controls, respectively. A representative experiment is shown. The experiment was repeated three times with similar results. *Indicates significant difference (Student’s *t*-test, *p* < 0.05) in luminescence between flg22 alone and flg22 + methanol/ethanol.

Another signaling step activated by many MAMPs and DAMPs is an oxidative burst, the rapid accumulation of ROS. We tested whether this oxidative burst would be altered in the presence of 3% methanol (**Figure 5A**) and ethanol (**Figure 5B**) in leaf disks of tomato. We found that systemin did not induce an oxidative burst (data not shown), while flg22 induced a strong oxidative burst, which had been reported before (Robatzek et al., 2007). Both methanol and ethanol alone were inactive, even at early time points. A double treatment of methanol or ethanol and flg22 induced a prolonged oxidative burst with higher ROS levels at later time points, as compared to flg22 alone. In double treatments, there was no significant difference in ROS accumulation over the first 20–30 min, but thereafter, ROS levels induced by the double-treatments were up to fourfold higher than in leaf disks treated with flg22 alone. This effect lasted until 40–50 min after treatment. Similar results (two of two experiments) were obtained with methanol and flg22 in the wild tomato species *S. cheesemaniae* (data not shown).

### METHANOL ALTERS FLG22- AND SYSTEMIN-INDUCED GENE EXPRESSION IN TOMATO LEAF TISSUE

**FIGURE 6:**
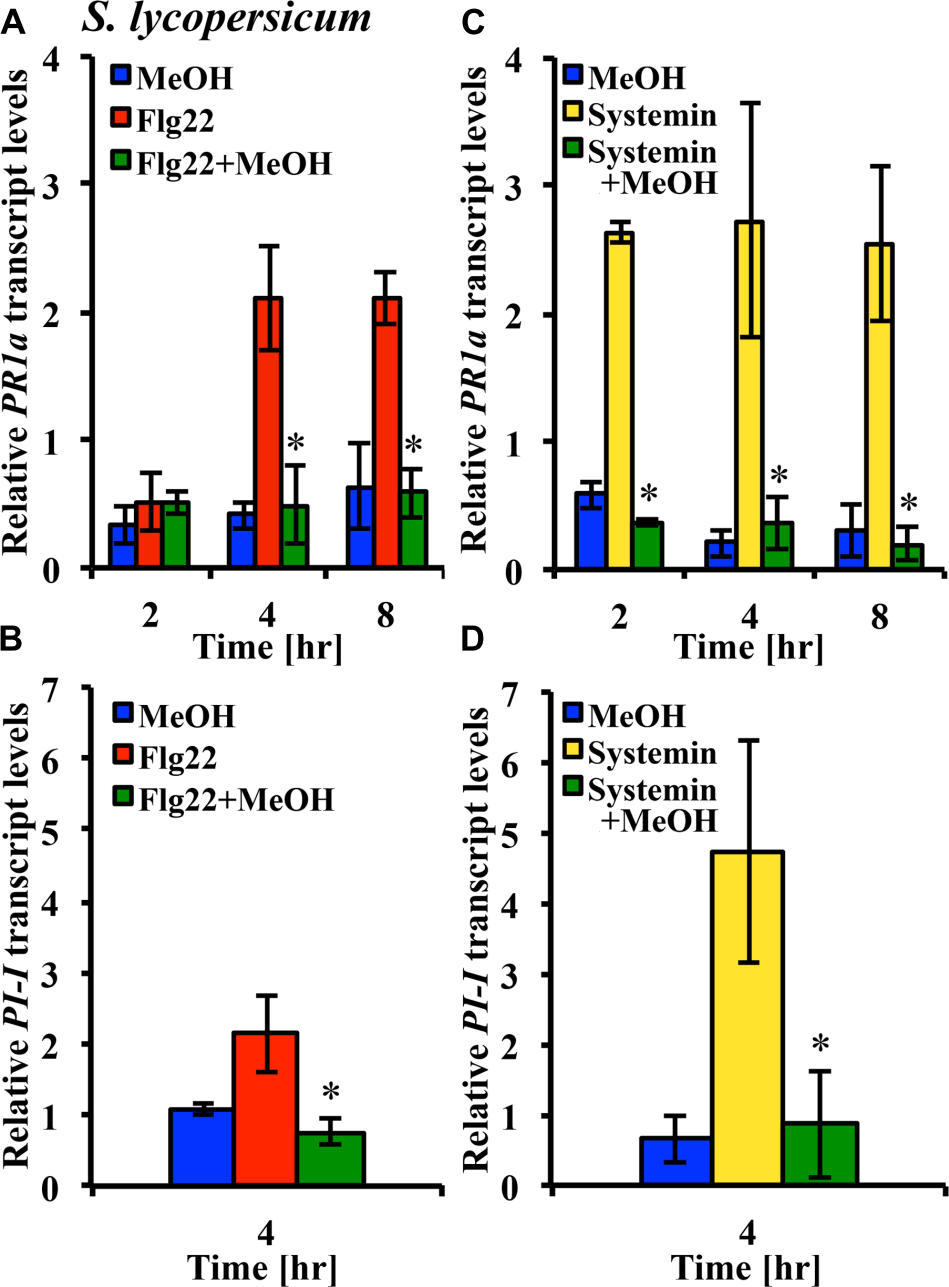
**Methanol suppresses flg22- and systemin-induced gene expression.** Tomato [*S. lycopersicum* var. Castlemart **(A,C)** or var. Rio Grande **(B,D)**] leaf disks were treated for 2, 4, or 8 h with water, 3% (v/v) methanol alone (blue bars), 25 nM systemin alone (yellow bars), 10 nM flg22 alone (red bars), or a combination of flg22 and methanol **(A,B)** or systemin and methanol **(C,D**; green bars). Expression of the defense genes *PR-1a* and *PI-1* was analyzed by quantitative RT-PCR. Bars represent the mean fold change ±SD in transcript levels above water controls from at least two independent experiments. *Indicates significant difference (Student’s *t*-test, *p* < 0.05) in gene expression between elicitor alone and elicitor + methanol.

Microbe-associated molecular patterns and ROS are components of plant signaling networks that transduce and relay stress signals to achieve output responses such as gene expression for proteins involved in defenses against herbivores and pathogens. We hypothesized that modulation of stress signaling by methanol would result in altered gene expression in response to DAMPs and MAMPs. We used the tomato leaf disk assay and quantitative RT-PCR to test whether methanol would alter the flg22- and systemin-induced expression of the *PR-1a* and *PI-1* defense genes in two tomato varieties. Methanol alone did not significantly induce expression of the two genes over a period of 8 h. In the Castlemart variety, flg22 and systemin induced upregulation of *PR-1a*, and this was completely suppressed by methanol over a period of 8 h (**Figures 6A,C**). In the Rio Grande variety, we only measured at 4 h and also found that methanol completely suppressed flg22- and systemin-induced upregulation of *PR1-a* (data not shown). We were unable to detect *PI-1* expression in Castlemart, but in Rio Grande, methanol completely suppressed systemin- and flg22-induced upregulation of *PI-1* at 4 h (**Figures 6B,D**). This shows that methanol alters flg22- and systemin-induced gene expression.

## DISCUSSION

### ARE METHANOL AND ETHANOL DAMPS?

Methanol is a volatile byproduct of PME activity and is rapidly released from plant cell wall material in response to pathogens and herbivory. Therefore, it is clearly damage-associated and a sign of the damaged self. If plants had a perception system for methanol that triggers defense responses, methanol would qualify as a DAMP elicitor. Consistent with this idea, Kang et al. (2011) had shown that methanol functions as an “elicitor” of defense gene expression in senescent rice leaves. However, we found that methanol does not function as a DAMP elicitor, but rather functions in a more subtle way, at least in dicots. In tomato, tobacco, and *Arabidopsis*, methanol alone has no pronounced elicitor activity, i.e., on its own, it does not induce an oxidative burst, activates MAPKs only very weakly and transiently, and did not induce defense gene expression over a period of 8 h. Other plant DAMPs like OGAs, systemins, extracellular ATP, and Peps function as strong elicitors of signaling and defense responses. With this regard, methanol is different. While it is damage-associated, its most profound effect is not elicitor activity, but altering the response to DAMPs and MAMPs.

Methanol is distinguished from other damaged-self signals by its chemical nature. Because of its chemical properties as the simplest alcohol, it may interact with plant cells in a different way as compared to other DAMPs. It has solvent properties and it decreases the dielectric constant of water. In a cellular microenvironment, this could affect the functioning of cell wall and plasma membrane-bound proteins as well as the integrity of the plasma membrane. These properties should also be pertinent to the methanol sensing mechanism, which could be either specific such as a specific sensor protein or receptor, or unspecific and based on cellular disturbances due to methanol effects on cellular or subcellular structures and macromolecules.

Furthermore, methanol can be metabolized to formaldehyde, formic acid, and eventually CO_2_ (Fall and Benson, 1996). These metabolites may have unique effects on plants such as toxicity (formaldehyde) or carbon metabolism (CO_2_). Methanol toxicity could also affect plant pathogens and herbivores. Transgenic tobacco plants overexpressing PMEs and producing much higher levels of methanol were more resistant against a wide range of herbivores. This was interpreted as a toxicity effect (Dixit et al., 2013).

In contrast to dicot plants, in darnel grass and tall fescue, methanol not only increases the MAPK response to a MAMP (chitosan) and wounding, but also functions as a relatively strong MAPK activator on its own.

The role of ethanol as a DAMP is not evident because its source is not clear. It can be produced under aerobic stress conditions (see Introduction), and it has been shown to be produced during special stages of pathogenesis, especially in plant interactions with soft-rot pathogens such as *Erwinia* species (Tolan and Finn, 1987), however, it was not investigated whether its generation requires a low oxygen environment. It is conceivable that hypoxia microenvironments are present in diseased aerial plant tissues, but this has not yet been investigated to our knowledge. Furthermore, herbivory of *Succisa pratensis* (Dipsacaceae) resulted in ethanol emissions, while unattacked *Succisa pratensis* plants did not emit ethanol. The source for this herbivory-induced ethanol remains to be determined (Peñuelas et al., 2005). Other evidence that suggest that ethanol may be produced in plants during stress, is stress-induced upregulation of plant alcohol dehydrogenase (ADH-P), which reduces acetaldehyde to ethanol. In general, ADH-Ps have been shown to play roles in scent production (reduction of C6-volatile hexenals to hexenols), as well as anaerobic and aerobic fermentation (Strommer, 2011). Upregulation of *ADH-P* genes has been shown not only for hypoxia, but also for abiotic stress conditions such as cold and dehydration (Dolferus et al., 1994). Furthermore, a range of stress signals (salicylic acid, UV light, *Phytophthora infestans*) upregulated an *ADH-P* gene in potato tubers and leaves (Matton et al., 1990) or during the common bean–bean dwarf mosaic virus interaction, which also leads to upregulation of pyruvate decarboxylase (Seo et al., 2007). The *Arabidopsis* eFP Browser () shows ADH1 upregulation in aerial plant tissue under abiotic stresses, including hypoxia, cold, osmotic, and salt, as well as in response to some pathogens.

If ethanol is produced during these stress conditions but not under normal conditions, then it has the potential for functioning as an alarm chemical, possibly as a DAMP or MAMP, depending on its source. We found that ethanol did not function as a strong elicitor in dicots, but rather altered the MAPK and ROS response to flg22 and systemin, similar to methanol. In grasses, both ethanol and methanol acted as strong elicitors of MAPK activity. It remains to be determined whether ethanol changes MAMP- and DAMP-induced gene expression. Since ethanol, unlike methanol, is not known to be produced during normal plant growth conditions, plants do not have to be able to distinguish stress-induced ethanol from other sources of ethanol. Therefore, ethanol could be a more reliable indicator of danger.

### IS METHANOL PERCEPTION ADAPTIVE?

Our data indicate that methanol may function as a damaged-self signal in the angiosperms. Since methanol is released during wounding and herbivory, and many bacterial and fungal pathogens employ PMEs to break down the plant cell wall barrier, methanol perception should be an adaptive trait if it results in appropriate defense responses. Not many studies have addressed this question, and the existing ones come to different conclusion (Downie et al., 2004; von Dahl et al., 2006; Körner et al., 2009; Kang et al., 2011; Dorokhov et al., 2012; see Introduction).

We investigated whether methanol would alter elicitor-induced defense genes. The tomato *PR-1a* gene is responsive to systemin and flg22 (**Figure 6**). It is often used as a marker gene for SA-dependent pathogenesis-related defenses (Van Loon and Van Strien, 1999; Schaller et al., 2000; Hind et al., 2011). The *PI-1* gene was highly responsive to systemin, but less responsive to flg22 in our experiments. Proteinase inhibitors like *PI-1* are typical herbivory-related genes and markers for JA-dependent defenses (**Figure 6**; Li et al., 2003, 2004; Hind et al., 2011). However, MAMP responses are not regulated in a strict hormone-dependent fashion. In *Arabidopsis*, flg22 activates defense genes independent of the stress hormones JA, SA, and ethylene (Zipfel et al., 2004).

Since the kinetics of flg22- and systemin-induced MAPK activity were differentially altered by methanol (**Figures 4A,B**) and only flg22 induced an oxidative burst in tomato, we hypothesized that methanol would alter systemin- and flg22-induced expression of *PR-1a* and *PI-1* differentially. But we found that 3% methanol completely suppressed upregulation of both genes in response to systemin and flg22. Methanol alone did not activate the two defense genes. In agreement with our results, von Dahl et al. (2006) found that spraying *N. attenuata* plants with methanol suppressed accumulation of trypsin proteinase inhibitor protein and rendered plants more suceptible to *M. sexta* larvae. PR-1a and PI-1 contribute to resistance against pathogens and herbivores. If additional defense genes would also be suppressed by methanol this may decrease resistance. In fact, some necrotrophic pathogens co-opt plant PMEs to make the plant cell wall more amenable to degradation by their pectic enzymes (Raiola et al., 2011). Susceptibility to these pathogens could be further increased by methanol via suppression of defense signaling pathways.

Our data are too limited to draw further conclusions about methanol effects on differential gene expression in response to MAMP/DAMPs. In a network as complex as the plant defense signaling network, output responses are difficult to predict based on signaling processes. A more comprehensive analysis of gene expression and signaling (including hormone signaling) will provide a more complete picture of how methanol shapes plant defenses and whether or in what context its perception as damaged-self is adaptive.

### DOES METHANOL SHAPE OUTPUT RESPONSES VIA MODULATION OF DAMP AND MAMP-INDUCED SIGNALING?

It is tempting to presume a causal relationship between altered gene expression and modulation of signaling in double-treatment experiments with elicitors and methanol. Our data only provide correlative evidence. Further studies are required to demonstrate a causal relationship, e.g., an analysis of methanol-induced gene expression in plants silenced for signaling genes such as MAPKs or ROS-generating NADPH oxidases, and genes involved in hormone signaling.

In dicots, we observed either a very weak methanol-induced MAPK activity (*S. peruvianum*, *N. tabacum*) or none at all (*A. thaliana*). On the other hand, methanol had a pronounced effect on MAMP/DAMP-induced MAPK activation and oxidative burst. With regard to amplitude of MAPK activity and ROS accumulation, the response to dual treatments was often synergistic, i.e., stronger than the sum of the responses to methanol and elicitor alone. In addition, methanol changed the MAMP/DAMP-induced MAPK activation kinetics, and it changed the activation spectrum (kinetics and amplitude) of three different active MAPKs in *Arabidopsis*.

We hypothesize that modulation of elicitor signaling by methanol can change the signaling outcome. This is supported by a classical study that determined the effects of nerve and epidermal growth factors on mammalian PC12 cells, which induce either differentiation or proliferation of the PC12 cells, respectively. Cell differentiation requires prolonged activity of the MAPKs ERK1 and ERK2, whereas proliferation requires transient activation of the same MAPKs. The difference in MAPK activation kinetics are brought about by specific growth factor receptor signaling, MAPK phosphatases that downregulate MAPK activity, and assembly of stable or short-lived signaling complexes (Kao et al., 2001; Pouyssegur and Lenormand, 2003). Manipulating MAPK activation kinetics has also been shown to have profound consequences for plant defenses. Transgenic plants that exhibit prolonged and stronger activation of MAPKs than control plants, synthesized higher amounts of ROS and developed hypersensitive response cell death (Zhang and Klessig, 2001; Ren et al., 2002, 2006). Prolonged MPK6 activation has also been correlated with effector-triggered immunity, whereas MAMP-triggered immunity resulted in transient MPK6 activation (Tsuda and Katagiri, 2010). Most MAMPs and DAMPs activate not only MAPKs and an oxidative burst, which are interconnected in a complex and signal-dependent manner (Pitzschke and Hirt, 2006, 2009), but also other kinases and phosphatases, ethylene and JA synthesis, and ion fluxes (Ca^2+^, K^+^, H^+^) across membranes (Ranf et al., 2011). This may explain overlaps in gene expression profiles induced by various MAMPs and DAMPs. However, the differences in the signaling profile can also explain elicitor-specific output responses (Denoux et al., 2008). Moreover, a combination of two or more different elicitors has the potential to alter the signaling profile of any single elicitor and thus the output (Holley et al., 2003; Higgins et al., 2007; Aslam et al., 2009; Schmelz et al., 2009; Atkinson and Urwin, 2012). In summary, it is possible that the altered gene expression response in our double treatment experiments is a consequence of modulation of elicitor-induced signal transduction by methanol.

In grasses, methanol alone has a strong effect on MAPK activity, even at relatively low concentrations (0.5–3%). Activation of MAPKs generally results in output responses such as gene expression or modification of enzyme activity (Liu and Zhang, 2004; Kandoth et al., 2007). Therefore, in grasses, methanol is likely to function as a typical DAMP elicitor.

### CAN PLANTS SPECIFICALLY RECOGNIZE DANGER-ASSOCIATED METHANOL?

Since methanol is not exclusively produced during pathogenesis and herbivory, a threshold sensing mechanism must be present that distinguishes damage-associated methanol from development- or steady-state-associated methanol, and translate this into a specific signaling response. This is also true for other damaged-self signals like oligogalacturonides. The activation of plant PMEs by wounding represents a mechanism that increases methanol emissions above steady-state levels (Körner et al., 2009). Our data support the idea of threshold sensing for the interaction of methanol and flg22 in *S. peruvianum* cells (observed in 60% of the experiments). We also observed synergism between methanol and systemin, however, this was independent of the methanol concentrations tested. Data for chitosan and methanol were too variable to draw conclusions on threshold levels. It is possible that concentrations of methanol above 3% are required to generate a more robust synergism with flg22 and chitosan. It is not known what methanol concentrations are reached in apoplast microenvironments of optimally growing plants and plants under attack by pathogens and herbivores, and to how much methanol sensing mechanisms are exposed before it is released through the stomata. In planta, methanol concentrations may exceed background levels in a highly local manner in cell wall or tissue microenvironments at attack sites. This would be exceedingly difficult to measure. Our data for ethanol in combination with flg22 and chitosan also showed a concentration dependent synergistic effect, whereas even the lowest ethanol concentration (0.3%) acted synergistically with systemin. Systemin signaling appears to be highly sensitive to both methanol and ethanol.

### IS METHANOL A PRIMARY DANGER SIGNAL?

It was proposed that damaged-self signals are primary signals that shape the response to more specific secondary elicitors from pathogens and herbivores (MAMPs and HAMPs; Heil, 2012). Since perception of DAMPs, MAMPs, and HAMPs results in rapid (within minutes) signaling responses, it is very difficult to determine whether a signal is a primary or secondary signal. For this a high resolution series of signaling events during an attack must be determined. Wounding of tomato seedlings by herbivores rapidly activates MAPK signaling within less than 3 min, locally and systemically (Stratmann and Ryan, 1997; Kandoth et al., 2007), which includes perception of an inducing signal and signal transduction down to the MAPKs. Because of the rapidity of the response, it was assumed that rapidly propagated electrical or hydraulic signals, volatile compounds, or ROS waves are the primary signals (Delano-Frier et al., 2013). However, some DAMPs will also be rapidly released when cells are macerated by the mandibles of chewing insects. A non-volatile DAMP signal alone is unlikely to upregulate a full defense response, as it is probably consumed by the feeding herbivore (Schittko et al., 2000). Only if the DAMP can trigger a rapid systemic response that can escape the herbivore, can the ringing of the alarm bell be heard. With this regard, the volatile methanol may serve as a primary damaged-self signal that can escape ingestion by herbivores and trigger or modulate secondary signaling responses. In the case of pathogens, the series of events is also not clear. Is the production of DAMPs by cell wall degrading enzymes or cell lysis the first event that activates plant defense signaling, or is it a MAMP like flg22 that plants perceive with the highly sensitive flg22 receptor FLS2 (Gomez-Gomez and Boller, 2000)? Methanol could function both as a primary and secondary signal which is rapidly produced by pathogen and plant PMEs. It could be released in two distinct phases, first during initial attack as a consequence of the action of pathogen PMEs, and second, during the defense response of the plant that includes increased PME activity to fortify cell walls via demethylesterification of pectin.

Heil (2012) further stated that most herbivore elicitors have not been tested without generating a wound site for elicitor application, which would release DAMPs. Therefore, it is possible that some elicitors only function in the presence of DAMPs. Even using suspension-cultured cells or protoplasts that express the appropriate receptor for a given elicitor can not rule out the presence of DAMPs during elicitor application. In *S. peruvianum* cells and protoplasts, chitosan, flg22, and systemin induce a strong signaling response (Hind et al., 2010), however, it can not be excluded that cell wall fragments and other DAMPs are present in the culture medium. At least, using suspension-cultured cells, we were able to test the activity of methanol without inflicting major damage to the cells.

## CONCLUSION

Our results on the function of methanol in plant defenses fit with the concept of the “damaged self” proposed by Martin Heil (Heil, 2009), albeit with modifications. Heil stated that “‘damaged-self’ signals should be taxonomically widespread, elicited by generalist herbivores and induce responses that act against many types of herbivore.” This concept can be expanded to include pathogens. Methanol emissions originate from plant cell wall pectin and are associated with damage inflicted by herbivores and pathogens. We showed that methanol perception by plants is “taxonomically widespread” in dicots and monocots. Furthermore, it may be an evolutionary ancient damaged-self signal. Evolution of PME and other pectin-related genes reflects the emergence of pectin-containing cell walls in the Charophytes, the closest green algal (Chlorophyta) group sharing a common ancestor with land plants (Popper et al., 2011; Sørensen et al., 2011; Wang et al., 2013; McCarthy et al., 2014). It is not known whether early plant or algal PMEs were involved in plant defenses, however, it is likely that fungal and bacterial PMEs co-evolved with pectin in land plants and their algal ancestors. We also showed that methanol is not a strong elicitor on its own, at least in dicots, but that it rather modulates elicitor-induced defense signaling and gene expression. This adds another level of complexity to the concept of the damaged-self. It remains to be determined whether it actually “acts against” pathogens and herbivores, or whether it shapes defenses in a more complex way.

## AUTHOR CONTRIBUTIONS

Claire T. Hann, James E. Dombrowski, and Johannes W. Stratmann conceived the project and designed the research; Claire T. Hann and Carlton J. Bequette performed all tomato, tobacco, and *Arabidopsis* experiments and contributed to writing the manuscript. James E. Dombrowski performed all grass experiments and wrote the respective parts of the manuscript. All authors analyzed the data. Johannes W. Stratmann supervised the project and wrote the manuscript.

## Conflict of Interest Statement

The authors declare that the research was conducted in the absence of any commercial or financial relationships that could be construed as a potential conflict of interest.
